# Comparing a Digital Health Check With Traditional Nurse-Led Health Examinations Among Long-Term Unemployed Individuals: Comparison Study

**DOI:** 10.2196/49802

**Published:** 2024-10-16

**Authors:** Venla Raussi, Sari Kujala, Iiris Hörhammer, Kaisa Savolainen, Reija Autio, Tuomas Koskela

**Affiliations:** 1 Faculty of Medicine and Health Technology Tampere University Tampere Finland; 2 Department of Computer Science Aalto University Helsinki Finland; 3 Faculty of Social Sciences (Health Sciences) Tampere University Tampere Finland; 4 Department of General Practice Faculty of Medicine and Health Technology Tampere University Tampere Finland; 5 The Wellbeing Services County of Pirkanmaa Tampere Finland

**Keywords:** chronic illnesses, eHealth, health care services, lifestyle, long-term unemployment, digital health check, primary prevention, risk assessment, risk factors, prevention, screening

## Abstract

**Background:**

A digital health check can be used to screen health behavior risks in the population, help health care professionals with standardized risk estimation for their patients, and motivate a patient to change unhealthy behaviors. Long-term unemployed individuals comprise a particular subgroup with an increased risk of lifestyle-related diseases.

**Objective:**

This study aims to investigate the clinical utility of a general digital health examination, the STAR Duodecim Health Check and Coaching Program (STAR), which was developed in Finland, in the targeted screening of long-term unemployed individuals. For this purpose, we compared health challenges identified by a digital health check with those identified by a nurse during a face-to-face health check for unemployed individuals.

**Methods:**

In this comparison study, 49 unemployed participants attending a health check were recruited from two Finnish primary health care centers. The participants used STAR and attended a nurse’s health check. Data were collected by surveys with multiple-choice and open-ended questions from the participants, nurses, and a study assistant who observed the session. The nurses were asked to name the three most significant health challenges for each participant. These health challenges were categorized into health challenges corresponding to STAR and these were compared with each other. Percentages of agreement between STAR and nurses were calculated. Sensitivity and specificity, as well as Cohen κ with *P* values and CIs, were computed for agreement.

**Results:**

STAR identified a total of 365 health challenges, an average of 7.4 (SD 2.5) health challenges per participant (n=49). The nurses named a total of 160 health challenges (n=47). In 53% (95% CI 38.1-67.9; n=25) of cases, STAR identified all categorized health challenges named by nurses. In 64% (95% CI 48.5-77.3; n=30) of cases, STAR identified at least 2/3 of the health challenges identified by nurses. Cohen κ was 0.877 (*P*<.001) for alcohol, indicating almost perfect agreement, and 0.440 (*P*<.001) for smoking and 0.457 (*P*=.001) for cholesterol, indicating moderate agreement. STAR left a total of 89 health challenges, an average of 1.8 (SD 1.1) per participant, uncategorized because STAR lacked an answer to the question or questions required for the classification of a certain health challenge. The participants did not always add information on their blood pressure (n=36, 74%), cholesterol (n=22, 45%), and waist circumference (n=15, 31%).

**Conclusions:**

In conclusion, STAR identified most of the health challenges identified by nurses but missed some essential ones. Participants did not have information on measurements, such as blood pressure and cholesterol values, which are pivotal to STAR in assessing cardiovascular risks. Using the tool for screening or as a part of a traditional health check with necessary measurements and dialog with health care professionals may improve the risk assessments and streamline the health checks of unemployed individuals.

**International Registered Report Identifier (IRRID):**

RR2-10.2196/27668

## Introduction

### Background

Long-term illnesses and multimorbidity have become more common, thus reducing quality of life and increasing the demand for health care services [1,2]. Lifestyle choices have a significant impact on the expected onset of diseases, age of death, risk factors concerning long-term illnesses, and multimorbidity [1,3-5]. Preventable lifestyle-related risk factors affecting chronic morbidity and mortality have been recognized, most notably smoking, the harmful use of alcohol, physical inactivity, and an unhealthy diet [5,6].

So-called eHealth uses digital information and communication technologies for health, demonstrating the growing potential to make health services more accessible, efficient, and cost-effective [7,8]. Digital health checks, an eHealth tool aimed at assessing lifestyle-related risk factors, could improve primary prevention in health care [9-11]. A digital health check can be used to screen health behavior risks in the population, help health care professionals with standardized risk estimation for their patients, and motivate a patient to change unhealthy behaviors [9]. Web-based interventions focusing on health behavior-related risks have been reported to have an overall positive effect on the user’s health, resulting in positive behavior changes [11-13]. Assessing multiple lifestyle-related risks at the same time provides an opportunity to review one’s health comprehensively and target multiple health-related risk behaviors simultaneously [9]. Such interventions have been well-received by patients compared to interventions targeting only one health-related behavior [9,12-14].

The STAR Duodecim Health Check and Coaching Program (hereafter, STAR) is a general digital health examination developed by Duodecim Publishing Company Ltd and the Finnish Institute of Health and Welfare [15-17]. The abbreviation STAR comes from the Finnish word for a digital health check. STAR gives users a report that includes an evaluation of their life expectancy and an estimated risk for developing common long-term illnesses based on questions about personal characteristics, health information, lifestyle, mental well-being, and relationships. In addition, STAR provides coaching courses from which users can choose the most suitable based on STAR’s recommendations. Thus, STAR provides tools for further improvement and long-term tracking of health. Users can set personal goals, participate in coaching, and follow up on the development of their health over time. STAR and its report are further described in the study protocol and Multimedia Appendix 1 [18]. Previous studies of STAR have mainly focused on creating a persuasive system design [18-21].

STAR’s life expectancy evaluation and risk evaluations are based on previous Finnish studies, namely the Finriski, Autoklinikka, and Minisuomi studies [15,22-24]. The information provided by risk calculators can help health care professionals identify risk categories more accurately and improve the likelihood of prescribing medicine to high-risk patients, thereby helping with decision-making [25]. On the other hand, there is a huge variety of health risk calculators available digitally. A systematic review of digital cardiovascular disease risk calculators found wide variation in risk assessment models, risk presentation, and results [26]. This study also found the risk calculators to have overall poor actionability, and that the available risk calculators often lack clinical validity [26].

Long-term unemployed individuals comprise a particular subgroup with an increased risk of lifestyle-related diseases [27]. Long-term unemployment is linked to greater than average morbidity, earlier expected age of death, and increased risk of mortality [27,28]. Long-term unemployment is defined as having been unemployed for 12 months or more [29]. The duration of unemployment increases the burden of disease [30]. Unemployment also affects self-assessed health negatively, and the strongest association is found in people with a lower socioeconomic status, weak social networks, and health-related reasons for unemployment [31]. There is also some evidence that unemployed people use preventive services less in Finland [32]. There have been studies on digital health checks and internet-based risk assessments of subgroups, such as the employed, but there have been few studies focusing on digital health checks for unemployed individuals [33].

Although the general health checks for the unselected population may not be cost-effective in reducing illness and mortality [34], targeted screening could be useful if it leads to action (by unemployed individuals or by the service system) [32]. The lack of benefit of health checks may be due to the fact that those who would need them the most do not participate in them [34]. Furthermore, there is not enough evidence on the potential clinical utility of digital health checks in targeted health risk screening. While digital health checks performed by citizens themselves may hold potential for the low-cost screening of targeted populations, more evidence is needed regarding their ability to identify health risks in these subgroups.

### The Goal of This Study

This study aims to investigate the clinical utility of a digital health check in screening targeted at long-term unemployed individuals. For this purpose, we compare the health challenges identified by a digital health check with those identified by nurses during a face-to-face health check for unemployed individuals. We report the agreement and differences in health challenges identified by the digital health check and the nurse’s check.

## Methods

### Recruitment

The inclusion criteria for participation in the study were unemployment for at least 12 months, age older than 18 years, and participation in a health check for long-term unemployed persons. Finland has a public health care system organized and financed by welfare counties, and every resident is entitled to receive social, health, and medical services [35,36]. According to the health care law, welfare counties are obliged to organize health checks for unemployed individuals [37]. The purpose of these health checks is to promote health and support the ability to function and work [38]. The initiative for a health check can come from the unemployed person, unemployment services, or the municipal social welfare administration.

We recruited 49 participants in total: 45 from Tampere and 4 from Espoo. The characteristics of the health check participants are described in Table 1. Three nurses from Tampere and three nurses from Espoo health centers participated in the study. Two participants were excluded from the health challenge analysis due to a lack of data from the nurses. In these cases, the participants skipped the nurse’s health examination or the nurse did not fill out the professional questionnaire. As a result, data were obtained from 47 participants.

**Table 1:** The characteristics of the health check participants.

Characteristics			Participants (N=49)
Age (in years), mean (SD)			47.63 (10.38)
**Sex, n (%)**			
	Male	28 (57)	
	Female	21 (43)	
Unemployment time in months, mean (SD)^a^			43.6 (55.28)
**What did you do before you became unemployed?, n (%)**			
	Studying	6 (12)	
	On sick leave	3 (6)	
	Working	32 (65)	
	Something else	8 (16)	
**Highest education, n (%)**			
	Elementary school	9 (18)	
	Vocational education	23 (47)	
	Upper secondary education	9 (18)	
	Bachelor’s degree	5 (10)	
	Master’s degree	3 (6)	
**Do you have a long-term illness diagnosed by a doctor?, n (%)^b^**			
	No	15 (31)	
	Yes	29 (59)	
	Don’t know	6 (12)	

^a^Information is missing from three (3).

^b^One (1) answered both yes and I don't know.

### Procedure

The recruitment process started when the health center assistant (or in Espoo, a nurse) booked an appointment for an unemployed person to nurse’s health check and told them about the possibility of participating in this study. Those who expressed their willingness to participate in the study were scheduled for a health check on the research day.

The participants attended a health check for unemployed individuals at the local health center, gave their consent on a consent form, and filled out participant questionnaire 1. Each participant filled out STAR and read its report while a study assistant observed and filled out the observer’s questionnaire. After reading the report, the participant filled out participant questionnaire 2. Next, the participant was directed to the nurse’s health check. After the nurse’s health check, the nurse filled out the professional questionnaire 1 before reading the STAR report. In professional questionnaire 1, the nurse was asked to name the participant’s three most significant health challenges. Then the nurse read the STAR report and filled out professional questionnaire 2. A flowchart of the recruitment process and the content of the questionnaires is described in more detail in the study protocol [18].

The initial idea was that the order of STAR and the nurse’s health check would be reversed after every 10 checkups [18] but implementing this proved difficult due to the nurses’ schedules, hence only a few cases (n=3) were assessed in reverse order.

The recruitment took place at two Finnish public health centers in Espoo and Tampere. Espoo and Tampere are the second and third largest cities in Finland, with a population of 290,000 and 240,000, respectively [39].

After completing STAR, the participant received a report from STAR including a list of personal health challenges identified from the answers. The term “health challenge” means a medical condition, disease, habit, or lifestyle that poses the risk of disease or medical ailment. In the STAR report, health challenges are categorized into red, yellow, and green categories. STAR defines the categories as follows: red=“Please check—this is essential for your health”; yellow=“Please check and pay attention”; and green=“Great, continue with the same pattern.” The STAR classification is based on the patient’s answers to the STAR questions. According to the limit values, STAR classifies a total of 17 different health challenges into these aforementioned color-coded categories. The 17 health challenges are BMI, waist circumference, exercise, diet, sleep, stress, mental resources, community action, alcohol, smoking, cholesterol, diabetes, blood pressure, oral health, ability to work, relationships, and family.

The total number of STAR health challenges per participant is not limited; STAR can categorize any number of the 17 different health challenges into red or yellow health challenges. In contrast, a nurse was asked to name only the three most significant health challenges in the questionnaire in this study.

If the user has not answered a question or questions about a specific health challenge, STAR cannot classify it into any of the previous color-coded categories. In this case, this health challenge appears as a gray category in the STAR report. STAR announces to the user that there is no classification information for the health challenge in question, and STAR cannot take a position on it in this case.

### Analysis Methods

The three top health challenges named by the nurse in the health check were compared to the health challenges found in the STAR report for the same person. These three health challenges named by the nurse were first classified according to the corresponding 17 categories in the STAR report so that they could be compared. Health challenges named by the nurse that could not be categorized into the same categories as in STAR remained uncategorized. They are referred to hereafter as “uncategorized.”

We calculated the cases in which STAR identified all the same health challenges as nurses did and the cases in which STAR identified at least 2/3 of the health challenges determined by the nurses. We also report the top four health challenges named by the nurse and STAR, respectively, the new health challenges STAR found, and any health challenges missed. We excluded work ability from the analyses because STAR does not classify work ability if the participant has answered that he or she is unemployed. In addition, we calculated for each health challenge the accuracy, sensitivity, specificity, and agreement with Cohen κ value with CIs and *P* value [40]. We performed a power analysis to determine the sample size necessary for detecting a moderate or higher agreement in our study. The analysis showed that 47 participants per condition would provide sufficient power (80%) to identify this agreement with a significance level of 0.05. We used R (version 4.0.1; R Core Team) [41] with the packages caret [42] and irr [43] for computing the sensitivity, specificity, accuracy, and κ values. In all statistical analyses, we considered *P* values <.05 to be statistically significant.

### Ethical Considerations

This study was approved by the Ethics Committee of the Expert Responsibility Area of Tampere University Hospital in June 2020 (ETL Code R20067). All participants signed the written informed consent form to take part in the study. They were informed about the purpose of the research, the expected duration of their participation, that their participation was voluntary, and that they could discontinue it at any time without it causing them any harm. Participants’ consent forms and all questionnaires collected in the study were archived. All questionnaires collected in the study were anonymized with numerical codes. Participants were not reimbursed and did not receive compensation for participating in the study.

## Results

### Overview

**Figure 1:**
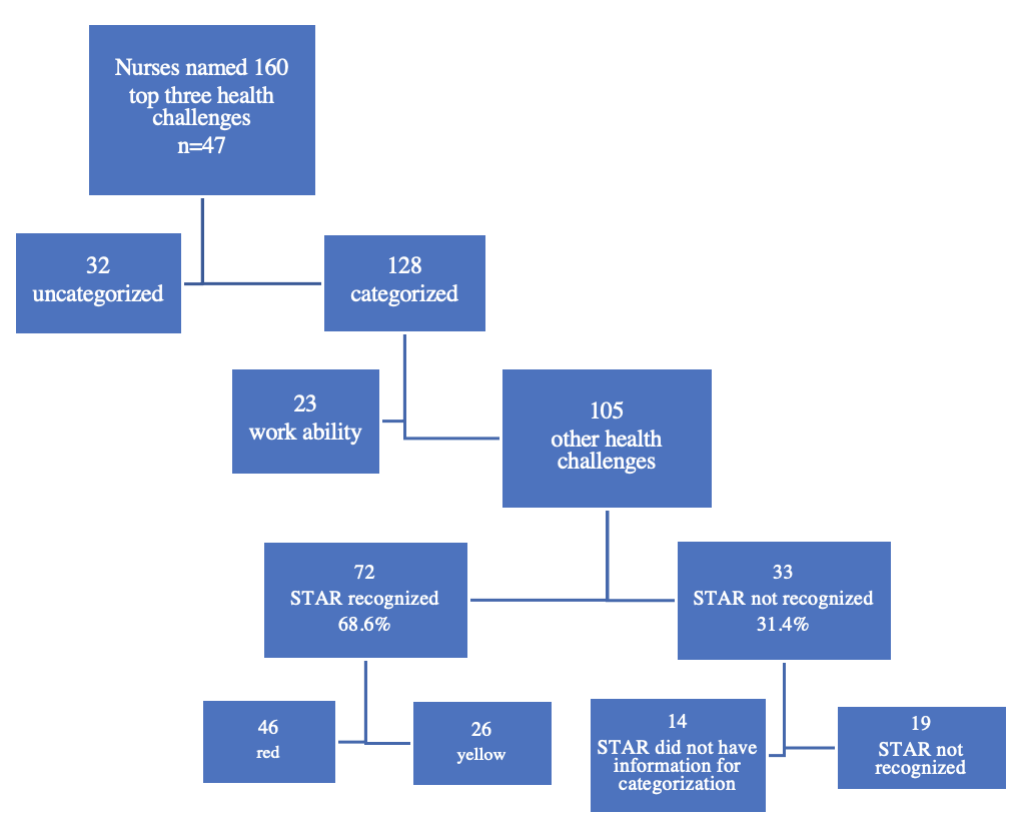
Flowchart of the different health challenges identified by the nurses and the digital health check (STAR) in the study.

STAR predicted an average life expectancy of 78.1 (SD 6.8) years for the participants. STAR identified a total of 365 health challenges (n=49), an average of 7.4 (SD 2.5) health challenges per participant (Table 2). Nurses named a total of 160 top 3 significant health challenges (n=47; Table 2). Of these, 128 health challenges determined by the nurses were categorized into the same 17 categories as in the STAR report (Table 2 and Figure 1). The remaining 32 health challenges named by the nurses could not be categorized in the STAR categories (Table 2 and Figure 1). STAR left a total of 89 health challenges, an average of 1.8 (SD 1.1) per participant, uncategorized in the color-coded categories because STAR lacked an answer to the question or questions required for classification for a certain health challenge (Table 2).

In 53% (n=25) of cases, STAR identified all categorized health challenges named by nurses (95% CI 38.1-67.9). In 64% (n=30) of cases, STAR identified at least 2/3 of the health challenges identified by the nurse (95% CI 48.5-77.3).

Nurses most often named mental resources (n=27, 57%), blood pressure (n=14, 30 I made the requested changes.), cholesterol (n=13, 28%), and BMI (n=10, 21%) as health challenges. STAR most often named diet (n=45, 96%), community action (n=38, 81%), BMI (n=34, 72%), and mental resources (n=32, 68%) as health challenges. Community action and diet were seldomly named in the top three health challenges by the nurses. Nurses did not name waist circumference, relationship, or family among the health challenges (Table 3).

**Table 2:** Characteristics of the health challenges named by STAR and the nurses.

	STAR (n=49)	Nurses (n=47)
Health challenges, number (mean, SD)	365 (7.4, 2.5)	160 (3.4, 0.9)
STAR red^a^, number (mean, SD)	189 (3.9, 2.3)	N/A^b^
STAR yellow^c^, number (mean, SD)	176 (3.6, 1.7)	N/A
Nurses’ categorized^d^, number (mean, SD)	N/A	128 (2.7, 1.1)
Nurses’ uncategorized^d^, number (mean, SD)	N/A	32 (0.7, 0.8)
STAR uncategorized^d^, number (mean, SD)	89 (1.8, 1.1)	N/A

^a^Red: Please check—this is essential for your health.

^b^N/A: not applicable

^c^Yellow: Please check and pay attention.

^d^Able to categorize according to the corresponding 17 categories in the STAR report.

**Table 3:** The matches of the health challenges in the nurse’s and STAR’s health checks (N=47).

	Nurse named	STAR named	Accuracy^a^	Sensitivity	Specificity	Cohen κ (95% CI)	*P* value (κ)
BMI	10	34	0.404	0.8	0.297	0.052 (–0.105 to 0.208)	.54
Waist circumference	0	23	0.50	N/A^b^	N/A	N/A	N/A
Exercise	6	20	0.617	0.667	0.610	0.138 (–0.082 to 0.359)	.20
Diet	7	45	0.191	1	0.050	0.015 (–0.009 to 0.039)	.55
Sleep	5	17	0.660	0.600	0.667	0.130 (–0.106 to 0.366)	.24
Stress	3	28	0.468	1	0.432	0.088 (–0.012 to 0.189)	.14
Mental resources	27	32	0.638	0.778	0.450	0.235 (–0.041 to 0.512)	.09
Community action	2	38	0.191	0.5	0.178	–0.034 (–0.12 to 0.053)	.26
Alcohol	4	5	0.979	1	0.977	0.877 (0.641 to 1)	<.001
Smoking	7	18	0.766	1	0.725	0.440 (0.2 to 0.68)	<.001
Cholesterol	13	8	0.809	0.462	0.941	0.457 (0.166 to 0.748)	.001
Diabetes	6	17	0.681	0.667	0.683	0.196 (–0.056 to 0.448)	.09
Blood pressure	14	9	0.681	0.286	0.848	0.150 (–0.144 to 0.443)	.29
Oral health	1	17	0.617	0	0.630	–0.042 (–0.122 to 0.038)	.45
Relationship	0	14	0.702	N/A	N/A	N/A	N/A
Family	0	9	0.801	N/A	N/A	N/A	N/A
Total	105	334	0.608	0.686	0.595	0.1467 (0.09189 to 0.2016)	<.001

^a^Cases the nurse and STAR agreed (=both named or neither named a health challenge).

^b^Cannot be computed since nurses did not find any of the participants to have these conditions.

Of the 105 categorized top three health challenges named by the nurses, STAR recognized 72 (69%) challenges; 46 (64%) challenges as red and 26 (36%) challenges as yellow (Figure 1). STAR missed 33 (31%) of the 105 challenges categorized as the top three health challenges by the nurses; STAR did not recognize 19 health challenges named by the nurses, and it was not even possible for STAR to recognize 14 of the health challenges named by the nurses because STAR lacked an answer to the question or questions required for categorization (Figure 1). The 14 health challenges named by nurses that STAR could not recognize were mental resources (n=1), cholesterol (n=4), and blood pressure (n=9).

STAR and the nurses recognized alcohol (n=46, 98%), cholesterol (n=38, 81%), and smoking (n=36, 77%) as health challenges very similarly (Table 3). Participants were not able to respond to all questions due to a lack of knowledge of some measurement values, and this had an impact on STAR’s ability to recognize blood pressure and cholesterol, which affected the agreement between the nurses and STAR. Cohen κ was 0.877 (*P*<.001) for alcohol indicating almost perfect agreement and 0.440 (*P*<.001) for smoking and 0.457 (*P*=.001) for cholesterol indicating moderate agreement (Table 3).

STAR named a total of 262 new health challenges (127 red and 135 yellow) that were not named by the nurses. STAR most commonly named diet (15 times) and community action (35 times) as red health challenges, while the nurses did not name them among the three most significant health challenges.

STAR left a total of 89 health challenges, an average of 1.8 (SD 1.1) per participant, unassigned according to the color-coded categories because STAR lacked an answer to the question or questions required for classification to a certain health challenge (Table 2). This means that STAR does not analyze and categorize a health challenge into any color-coded category in the STAR report if it lacks the information necessary for classification, such as information about waist circumference or cholesterol values, as mentioned above. The participants did not most often add information about their blood pressure (n=36, 74%), cholesterol (n=22, 45%), and waist circumference (n=15, 31%). Additionally, 22.4% (n=22) of the participants had not filled in information about diabetes in STAR, while diet, stress, mental resources, relationships, and family were missing each from one participant (n=1, 2%).

### Health Challenges Identified by the Nurse That Could Not Be Categorized

The 32 health challenges named by the nurse that could not be classified into the 17 categories of the STAR report were not identified by STAR. Musculoskeletal diseases were the most common of them. The nurses have named musculoskeletal diseases 9 times among the health challenges, but STAR was unable to identify them. In addition, STAR does not classify work ability into 3 different color-coded categories if the participant has responded that he or she is not employed. However, the nurses mentioned work ability as a health challenge in 49% (n=23) of the participants, which caused a discrepancy (Figure 1). There were also some specific diseases and symptoms that STAR could not recognize, for example, migraine, chronic obstructive pulmonary disease, fibromyalgia, and impaired hearing. Nurses also mentioned various psychosocial problems among the health challenges, for example, autism and fear of social situations, which STAR was unable to identify. There were also some laboratory values, problems in taking care of the participant’s own health, and diseases that run in the family, which the nurses had mentioned, but STAR was unable to identify.

## Discussion

### Principal Results

STAR recognized the health challenges named by the nurse well if the particular health challenge was covered in the questions of STAR. In half of the cases, STAR identified all the health challenges identified by the nurses. Alcohol consumption, smoking issues, and elevated cholesterol levels in particular were recognized similarly. On the other hand, the existence of certain health challenges was often left unassessed by STAR due to a lack of input from the patients. The participants most often lacked information about their blood pressure, cholesterol, and waist circumference. Furthermore, musculoskeletal diseases were not covered in STAR questions and were not recognized as health challenges in the STAR report, but they were listed among the top three health challenges by the nurses. On the other hand, STAR sensitively named diet and community action among the health challenges.

The life expectancies of unemployed individuals in this study group (average 78.1, SD 6.8 years) did not differ substantially from the national average life expectancy at birth [44]. However, the life expectancies of the participants in the STAR reports were probably overestimated, as many participants lacked information on essential risk factors to be taken into account in estimations of their life expectancy. Above all, the majority lacked information on blood pressure. High blood pressure is known to be a significant factor in reduced life expectancy [45].

### Comparison With Prior Work

Evidence on the impacts of digital health checks is limited. Especially, there is no previous research on comparisons between digital health checks and nurse-led health examinations. However, in a feasibility study in England, it was found that digital health checks for reducing alcohol intake among employees appeared feasible and acceptable, but the study included low participation rates, potentially attracting “worried well” employees rather than those at greatest health risk [46]. Our study complements the perception that hazardous alcohol use can be identified well with digital health checks and our study increases the knowledge of the uniformity of the assessments of digital health checks and nurse examinations regarding the identification of alcohol use.

Furthermore, a scoping review of the usability and utility of eHealth for physical activity counseling in primary health care centers found technical problems and the complexity of programs to be notable usability barriers to eHealth [47]. In this study, the unemployed participants’ ignorance of their health-related parameters, such as blood pressure, cholesterol, and waist circumference, among other things, had a significant impact on STAR’s recommendations. In an Australian study, it was found that most web-based heart age calculator users did not know their cholesterol values but knew their blood pressure values [48]. However, in this study, differently, almost half of the participants did not know their cholesterol values and 3 of 4 participants did not know their blood pressure values.

### Strengths and Limitations

The strength of the study was the prospective design in a real-life setting focusing on potential high-risk people that could potentially benefit from the health check, and there is little previous research in regard to targeted screening among this particular group. In addition, as a reference for the digital health checks, we had the nurses’ assessments of the top three health challenges for the same participants.

The limitation of the study was the small number of participants, which may have had an impact on the results. It was slow to gather study material due to the tight schedules in the participating health centers. There was a shortage of nurses during the COVID-19 pandemic, which slowed down the data collection. Furthermore, in many cases motivating unemployed individuals to participate in this study was difficult, possibly due to their individual and complex situations and their low interest in digital applications. In addition, many of the participants did not show up for the health examination. However, the sample size was sufficient for statistics concerning moderate or higher levels of agreement between the two measures.

The nurses were requested to name only the top three health challenges; hence an exact comparison of the health challenges between the nurses and STAR was not possible. Furthermore, deviating from the instructions, some nurses named more than three health challenges per participant but this did not have an impact on agreement analyses. In addition, the use of a digital tool as an intervention may have had an impact on the participants’ thoughts about their health before the nurse’s visit. Furthermore, there is a risk of selection bias because people who are comfortable with digital tools will probably more readily agree to participate compared with those who are not familiar with such technology. People who struggle with computers and technology may have refused to participate, even though they could provide important information concerning usability and the user experience. It has been reported that the users of eHealth interventions are more likely to be highly educated and have a healthier lifestyle than average, while those who could benefit the most are not using them [49,50].

The health challenges named by the nurses may have differed from those in the STAR report due to differences in terminology. In addition, the interpretation of the open-answered terms was challenging. It proved difficult to classify the health challenges named by the nurses and STAR as being exactly correspondent.

### Implications

STAR recognized quite well the health challenges named by the nurses but missed some essential ones. This reflects the focus of STAR on cardiovascular risk factors, which seem to cover the health and well-being concerns of unemployed individuals only partially. In Finland, for instance, the greatest health challenges related to the subjective inability to work are musculoskeletal and mental disorders [51]. The nurses often named musculoskeletal complaints as health challenges, but STAR was unable to identify them. In the future, STAR could be developed to identify health challenges related to musculoskeletal health as well due to their commonness and impact on health and disability [52]. Furthermore, participants did not remember or did not have information on crucial cardiovascular risk factors (eg, blood pressure, cholesterol values, and waist circumference), which had an impact on risk assessments. It is obvious that STAR would better identify health challenges if the participants had answered all the questions in STAR and known all the values needed in the answers. In this case, the report would have been also more plausible and reliable for the participant. Before filling out the STAR health check, the important values should be reminded to the user. In addition, the focus of the nurse’s health check differs from the digital health check to some extent. STAR is focused on assessing cardiovascular risk and mental well-being; hence, a more comprehensive health-related assessment may have been missed in the digital health check. STAR did not identify specific diseases or symptoms, such as migraine, chronic obstructive pulmonary disease, and impaired hearing, because STAR is instead specially designed to identify risk factors associated with lifestyle, substance use, cardiovascular health, and mental well-being and the impact of various parameters (eg, blood pressure and cholesterol) on health and risk of illness.

STAR identified significantly more health challenges (an average of 7.4, SD 2.5, health challenges per participant) than the nurse was asked to name. The large number of health challenges named by STAR may be unmotivating from the participant’s point of view. Although STAR prioritizes health challenges by classifying them into red and yellow, STAR could provide a more precise prioritization to enable the user to know which of the health challenges would be the most important to fix. This could also help nurses to assess which health challenge is the most significant for the patient.

According to our results, STAR may be used as a screening tool before the nurse’s face-to-face health checks with unemployed individuals. This study does not give answers to the question of who needs a face-to-face health check in this group. However, it seems evident that a nurse’s health check covers various aspects, especially in complex cases. Face-to-face encounters and interactions may facilitate the discussion of patient-centered health issues, which may be missed in digital screening. However, a digital health check could help to identify in a structured manner the most relevant health issues and identify the patients whose needs are the greatest. This is in line with Nordic general practice core values and principles [53]. Our results reveal that an effort should be put into providing support for self-measuring health indicators essential for digital health checks.

### Conclusions

In conclusion, STAR identified most of the health challenges identified by the nurses but missed some essential ones. STAR is focused on assessing the risks of cardiovascular diseases and mental well-being, and some other aspects of health were missed. Users did not have information on crucial risk factors (eg, blood pressure and cholesterol values), which are pivotal in assessing cardiovascular risks. Using the tool for screening or as a part of a traditional health check with necessary measurements and dialog with health care professionals may improve the risk assessments and streamline health checks of unemployed individuals.

## Appendix

Multimedia Appendix 1 The questionnaires used in the study.

## Abbreviations

STAR: STAR Duodecim Health Check and Coaching Program

## Data Availability

The datasets generated and analyzed during this study are not publicly available due to limitations of ethical approval and privacy policy, but they are available from the corresponding author upon reasonable request.
